# Activation of Phospholipase C Mimics the Phase Shifting Effects of Light on Melatonin Rhythms in Retinal Photoreceptors

**DOI:** 10.1371/journal.pone.0083378

**Published:** 2013-12-26

**Authors:** Susan Semple-Rowland, Irina Madorsky, Susan Bolch, Jonathan Berry, W. Clay Smith

**Affiliations:** 1 Department of Neuroscience, University of Florida, Gainesville, Florida, United States of America; 2 Department of Ophthalmology, University of Florida, Gainesville, Florida, United States of America; Morehouse School of Medicine, United States of America

## Abstract

Many aspects of retinal photoreceptor function and physiology are regulated by the circadian clocks in these cells. It is well established that light is the primary stimulus that entrains these clocks; yet, the biochemical cascade(s) mediating light’s effects on these clocks remains unknown. This deficiency represents a significant gap in our fundamental understanding of photoreceptor signaling cascades and their functions. In this study, we utilized re-aggregated spheroid cultures prepared from embryonic chick retina to determine if activation of phospholipase C in photoreceptors in the absence of light can phase shift the melatonin secretion rhythms of these cells in a manner similar to that induced by light. We show that spheroid cultures rhythmically secrete melatonin and that these melatonin rhythms can be dynamically phase shifted by exposing the cultures to an appropriately timed light pulse. Importantly, we show that activation of phospholipase C using m-3M3FBS in the absence of light induces a phase delay in photoreceptor melatonin rhythms that mirrors that induced by light. The implication of this finding is that the light signaling cascade that entrains photoreceptor melatonin rhythms involves activation of phospholipase C.

## Introduction

Vertebrate retinal photoreceptor physiology is intimately linked to their circadian clocks [1]–[5]. These clocks ensure that gene expression [2], [6]–[8], outer segment renewal [9]–[11], cGMP-gated channel function [12], L-type voltage-gated calcium channel function [13], and melatonin synthesis and release [1], [14]–[16] within and across the retinal photoreceptor population are temporally synchronized to the 24 hour light/dark cycle. Light is the primary stimulus that entrains photoreceptor clocks. Given the impact that circadian clocks have on photoreceptor physiology, it is surprising that the biochemical cascade through which light entrains the clocks in these cells remains unknown.

There are clues about the nature of the biochemical cascade that mediates light entrainment of retinal photoreceptor cell clocks. Perhaps the strongest clue comes from a study of light entrainment of the circadian clocks in the photoreceptive pinealocytes of chickens [17]. The results of this study showed that the heterotrimeric G-protein G_11_ is able to interact with pinopsin, the pineal opsin protein expressed in these cells, in a light- and GTP-dependent manner, and that selective activation of G_11_ in the absence of light induces phase shifts in the oscillators in these cells that resemble those induced by light. Importantly, these authors also showed that G_11_ is expressed in chicken retinal photoreceptors, that this protein is associated with rhodopsin in the dark, and that it dissociates from light-activated rhodopsin in a GTP-dependent manner. G_11_ immunoreactivity has also been observed in the retinal photoreceptors of mice and cows [18].

If activation of G_11_ is required for light entrainment of the oscillators in retinal photoreceptors, then we would expect to find components of the signaling cascade triggered by activation of G_11_ in these cells. The canonical signaling cascade triggered by activation of G_11_ involves activation of phospholipase C (PLC) that leads to increases in production of inositol 1,4,5-triphosphate (IP_3_) and in cytosolic Ca^2+^ levels [19]. The fact that vertebrate photoreceptors express PLC [18], [20]–[24] and that light-dependent PLC activity has been detected in the rod outer segments of many species, including amphibians, mammals, and birds [23], [25]–[28] further supports a potential role for a G_11_ - PLC cascade in light entrainment of retinal photoreceptor clocks. Interestingly, recent studies of nonvisual light receptive cells in vertebrate retina suggest that G_q/11_ - PLC signaling may be a conserved entrainment cascade in vertebrates and invertebrates. There is now compelling evidence that light activation of melanopsin in intrinsically photoreceptive retinal ganglion cells (ipRGCs) activates a G_q/11_ - PLC cascade [29] that in chicken retina has been shown to alter rhythmic melatonin production by these cells [30], [31].

The overarching hypothesis that guides our investigations of photoreceptor circadian biology is that the biochemical cascade that mediates light’s effects onthe circadian clocks in retinal photoreceptors involves activation of a G_q/11_-PLC signaling cascade. In this series of experiments, we set out to test the hypothesis that activation of PLC in photoreceptors in the absence of light can induce a phase shift in the melatonin secretion rhythms of these cells that mirrors that induced by a similarly timed pulse of light. To test our hypothesis, we compared melatonin secretion rhythms in retinal re-aggregation cultures that were exposed to a 12 hour light, 12 hour dark (12L:12D) cycle and were then exposed to a 6 hr pulse of either light or PLC agonist that was initiated at zeitgeber time (ZT) 12 prior to moving the cultures to constant darkness. We predicted that activation of PLC would induce a phase delay in the melatonin rhythm similar to that induced by light.

## Materials and Methods

### Reagents

#### Pharmacological agents

m-3M3FBS (2,4,6-Trimethyl-*N*-[3-(trifluoromethyl)phenyl]benzenesulfonamide, Tocris) activates phospholipase C without preference for any particular isoform, functioning with an EC_50_ in the low micromolar range [32]. A 100 mM m-3M3FBS stock solution was prepared in 33% DMSO in water and was used at a final concentration of 10 µM in our experiments.

o-3M3FBS (2,4,6-Trimethyl-*N*-[2-(trifluoromethyl)phenyl]benzenesulfonamide, Tocris) is an inactive ortho-substituted version of the above PLC activator that does not stimulate PLC even at millimolar concentrations [32], and was prepared as described for m-3M3FBS.

#### Antibodies

CERN-901, a rabbit polyclonal antibody that was raised against purified chicken rhodopsin, was used at a dilution of 1∶2000 and was a gift from Dr. W. DeGrip, Nijmegen, The Netherlands.

CERN-906, a rabbit polyclonal antibody raised against purified chicken red and green cone pigments, was used at a dilution of 1∶30,000 and was a gift from W. DeGrip, Nijmegen, The Netherlands.

Mouse anti-visinin (7G4) was obtained from the Developmental Studies Hybridoma Bank, Iowa City, IA and was developed by Dr. C. Cepko.

Alexa Fluor 588 and 594 anti-rabbit and anti-mouse secondary antibodies were used at a dilution of 1∶500 and were obtained from Molecular Probes (Eugene, OR).

### Animals

Fertile chicken eggs (*Gallus domesticus*, Rhode Island Red) were obtained from our in-house breeding colony. All animal procedures were carried out in strict accordance with the recommendations in the Guide for the Care and Use of Laboratory Animals of the National Institutes of Health and were approved by the Institutional Animal Care and Use Committee at the University of Florida (project 201101464).

### Retinal Cultures

Retina re-aggregation cultures were prepared as previously described [33], [34] using retinas of embryonic day 6 (E6) chicken embryos under conditions that favored the formation of retinal spheroids [35]. The re-aggregated retinal cultures prepared under these conditions contain well-differentiated rod and cone photoreceptors that have been shown to express visual pigments by culture day 4 [36]. These cultures also express arylalkylamine-N-acetyltransferase and hydroxyindole-O-methyltransferase, and importantly, have been demonstrated to secrete melatonin in a light period-dependent manner beginning at culture day 7 [37]. Briefly, the eyes from E6 chicken embryos (1 embryo yielded 2 cultures) were isolated and kept on ice in a 35 mm Petri dish containing DMEM supplemented with 0.1% penicillin/streptomycin (Cellgro) until dissection. Micro-dissection scissors and forceps were used to remove the anterior segment of the eye including the peripheral regions of the neural retina, lens, and vitreous body. The central parts of retina were carefully separated from adhering connective tissue and all but approximately 10% of the retinal pigment epithelium was removed. All pieces of the dissected central retina were placed in 1 ml of tissue dissociation media (0.008% trypsin-EDTA, Gibco; 0.05 U/ml dispase II, Roche; and 1% w/v collagenase Type 3; Worthington in DMEM) and were incubated at 37°C for 10 minutes. Enzyme activity was stopped at the end of the incubation by adding 1 ml of aggregation media (10% heat inactivated fetal bovine serum, High Clone; 2% filtered chicken serum, Sigma; and 0.1% penicillin/streptomycin in DMEM-F12, Gibco).

The digested tissues and solution were transferred to a sterile 15 ml conical tube and additional culture media (5 ml) was added to the sample. After centrifugation for 5 minutes at 300×g, the supernatant was removed and the retinas were mechanically dissociated in 1 ml of fresh aggregation media by gently pipetting them 7–10 times through a 1 ml plastic micropipette tip. After dissociation, additional aggregation medium (5 ml) was added to the sample and the sample was centrifuged again for 5 min. The supernatant was removed and the cell pellet was resuspended in 6 ml of fresh aggregation media.

Six-well culture plates were prepared by placing 0.4 µm Millicell cell culture inserts (EMD Millipore) into each well. To set up the cultures, 1 ml of aggregation media was carefully pipetted into each well outside the insert and an additional 1.5 ml of aggregation media was pipetted into each of the inserts. Finally, 500 µl of retinal cell suspension was added to the 1.5 ml media in each insert. The cultures were incubated on an orbital shaker (Bellco) at ∼70 RPM that was placed inside a CO_2_ incubator maintained at 37°C and 5% CO_2_. The 12L:12D cycle within the incubator was generated using a Switch LED 40 bulb with a color temperature of 2700 K and light output of 450 lumens that was placed about 12 inches from the cultures and controlled by a 24 hr timing device. The cultures were maintained on a 12L:12D cycle prior to exposure of the cultures to the various treatments on day 7. During the first 5 days in culture, the cultures were fed every other day at a different time within the 12 hr light period by removing 2 ml of media from the cultures, 1 ml from inside and 1 ml from outside the culture insert, and adding 2 ml fresh media to the inside of the insert. This feeding schedule was adopted so that media exchange in and of itself would not provide a synchronizing stimulus for the photoreceptor oscillators. On day 6, when media collection was started for melatonin assays, 800 µl of media was removed from the outside of the culture insert and placed in a microfuge tube and 800 µl of fresh media was added to the inside of the insert at each of the sampling times. Samples collected during the dark period were collected under dim red illumination. All samples were frozen and kept at −20°C until assayed.

### Lighting and Drug Delivery Schedules

All experimental and control cultures were maintained on a 12L:12D cycle for 6 days prior to the start of the experiment. Experimental groups included cultures that were exposed to either a 6 hr pulse of light or 6 hr pulses of either 10 µM m-3M3FBS or 10 µM o-3M3FBS that were delivered at ZT12 on culture day 7. Following treatment, all cultures were kept in continuous darkness for the remainder of the experiment. The PLC activator, m-3M3FBS, has been reported to exhibit stable activity in buffered solution over a 24 hr period [32]. o-3M3FBS is an inactive form of this activator [32]. These pharmacological agents were delivered to the cultures by removing as much of the media from the cultures as possible and replacing it with 2 ml of fresh media containing 10 µM of the agent. At the end of the 6 hr treatment period, the media containing the agent was removed from the cultures and replaced with fresh media. Control cultures were exposed to the 12L:12D cycle for 6 days and then placed in continuous darkness.

### Melatonin Assay

Aliquots of the culture media were removed from each culture at ZT0 and 12 on culture day 6 and then every 6 hrs beginning at ZT0 on day 7 until the end of the experiment. The levels of secreted melatonin in the media samples were determined using a commercially available, competitive ELISA assay (IBL International, Hamburg, Germany) according to the manufacturer’s protocol. Briefly, melatonin was extracted from culture media with methanol on a C18 silica column (Waters Corp, Milford, MA). These samples were then assayed for melatonin using a competitive enzyme-linked absorption assay that employed biotinylated melatonin. The concentration of melatonin in the samples was determined by comparison to a melatonin standard curve (1 pg/ml – 1 µg/ml) that was generated using melatonin (Sigma-Aldrich; St. Louis, MO) dissolved in culture media. Control analyses of freshly prepared aggregation media showed that the media itself did not contain detectable levels of melatonin.The rate of melatonin secretion in a given culture was calculated by dividing the difference in the concentration between two sequential time points by the time elapsed between sampling, accounting for the 40% media volume that was replaced by fresh media after sampling. Relative melatonin secretion rates were then calculated by normalizing the secretion curves to the maximum melatonin secretion rate obtained for a given culture. The relative stability of melatonin in our culture system was examined by adding known amounts of synthetic melatonin (Sigma-Aldrich) to culture media and than measuring melatonin levels in these samples after they were incubated at 37°C on a 12L:12D cycle for nine days. The results of these analyses revealed that there were no detectable changes in melatonin in these media samples under our culture conditions (data not shown). In addition, we examined the effects of supplementation of our culture media with 100 µM 5-hydroxy-L-tryptophan, a precursor of serotonin that has previously been shown to enhance synthesis and release of melatonin in studies of cultured *Xenopus* retinas [38]. Unlike the 10–20-fold increase in levels of released melatonin observed in the *Xenopus* retina experiments, we only observed a modest increase (<20%) in secreted melatonin levels (data not shown), a result indicating that this melatonin precursor was not rate-limiting in our culture system. Thus, we discontinued supplementation of our culture media with 5-hydroxy-L-tryptophan.

### Data Analyses

Melatonin secretion rhythms were compared using two-way repeated measures ANOVA with groups and time as main factors, followed by one-way ANOVAs at each time point comparing groups. The culture groups compared in the two-way ANOVA were either untreated or were exposed to either a 6 hr pulse of light or a 6 hr pulse of either 10 µM m-3M3FBS or 10 µM o-3M3FBS. Significant differences obtained from the one-way ANOVAs (*ρ*<0.05) were followed up with Student-Newman-Keuls post-hoc tests. A *ρ-*value of less than 0.05 was considered significant. All analyses were carried out using IBM-SPSS version 21.

### Immunocytochemistry

Retinal spheroids were fixed by immersion in 4% paraformaldehyde in PBS for 30 min at room temperature. The fixed tissues were then sequentially incubated in 30% sucrose in PBS overnight at 4°C, and then in a solution containing 50% Tissue-Tek Optimum Cutting Temperature (OCT) compound (VWR Scientific) and 15% sucrose in PBS on a shaker for 6 hr at room temperature. The tissues were then frozen in OCT blocks, sectioned at 6 µm, air dried, and then either stored at −20°C until use or processed for immunostaining. Sections were washed 2×15 min at room temperature in PBS to remove the OCT, incubated in ice cold methanol for 3 min, and then incubated for 1 hr at room temperature in blocking solution (10% v/v normal goat serum and 0.1% Triton-X 100 in PBS). After blocking, the sections were incubated with primary antibody for 2 hr at room temperature, washed for 3×10 min with PBS, and then incubated for an additional 2 hr at room temperature in species appropriate secondary antibody. Finally, sections were washed (3×10 min with PBS) and then counterstained using Hoechst (10 mg/ml in water; Molecular Probes). All primary and secondary antibodies were diluted using blocking buffer.

## Results

### Melatonin Secretion Rhythms in Re-aggregated Retinal Cultures Exhibit Dynamic Light Entrainment

The maturation of photoreceptors in re-aggregated retinal cultures recapitulates many aspects of development observed in intact retinas, albeit with a slightly accelerated time course [39], [40]. These cultures have been shown to contain well-differentiated photoreceptors [36] that rhythmically secrete melatonin [37]. Before committing to use of this culture model in our circadian experiments, we wanted to first establish that our version of the culture system replicated Willbold et al. [37]. Specifically, we wanted to determine if our cultures contained differentiated photoreceptors that were rhythmically producing melatonin.

**Figure 1:**
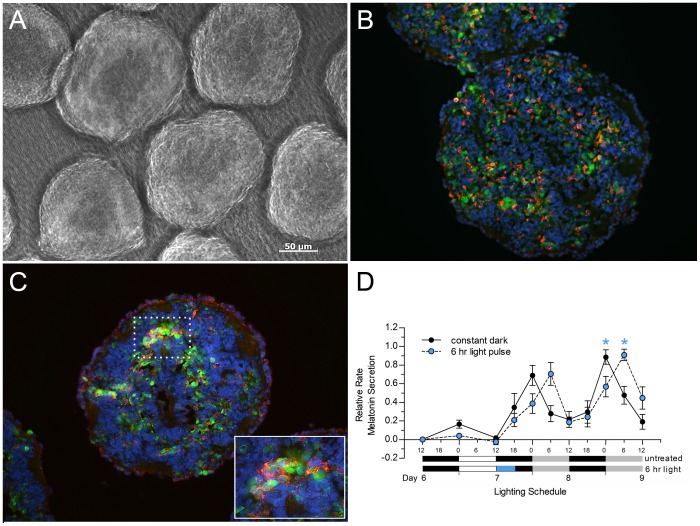
Photoreceptors and their melatonin rhythms in re-aggregate chicken retina cultures. A. Light microscopic image of re-aggregated, retinal spheroids grown for 12 days in culture on a Millicell membrane with continuous shaking. B,C. Sections of retinal spheroids cultured for 12 days and immunostained with an antibody against visinin (green) and either a chicken rhodopsin (CERN 901; red) (B) or a chicken red and green cone pigment (CERN 906; red) antibody (C). D. Melatonin secretion rhythms from re-aggregate cultures maintained on a 12L:12D cycle for the first six days of culture. On culture day 7, cultures were either placed in constant darkness at the beginning of the 12 hr dark period for the remainder of the experiment (black symbols), or were exposed to a pulse of light during the first 6 hrs of the 12 hr dark period (blue rectangle in lighting bar) and then placed in constant darkness for the remainder of the experiment (blue symbols). Levels of melatonin in the media were measured by competitive ELISA and were used to calculate the melatonin secretion rate from the cultures. Graphs show means +/− SEM (n = 6). Time points at which the one-way ANOVA post-hoc tests indicated significant differences (*ρ*<0.05) between the melatonin synthesis rhythms are indicated by blue asterisks. ONL – outer nuclear layer; INL – inner nuclear layer. Magnification bar = 50 µm.

To identify differentiated rod and cone cells in our cultures, we stained the re-aggregated retinal cultures that had been produced from E6 chicken retinas with antibodies directed against photoreceptor rod and cone visual pigments [33], [41] and visinin, a cone-specific Ca^2+^-binding protein expressed in chicken photoreceptors [42]. After 12 days in culture, the dispersed retinal cells had formed several spheres, all of which were approximately 200 µm in diameter (Fig. 1A). Sections of the spheres co-stained with the rod opsin (CERN 901) and visinin (cone-specific) antibodies revealed that numerous clusters of rods and cones were distributed throughout the spheres (Fig. 1B), a distribution pattern resembling that previously reported for re-aggregated cultures grown in the absence of retinal pigment epithelium [40]. Staining of our spheres with antibodies directed against red/green cone pigments (CERN 906) and visinin resulted in co-labeling of many cone photoreceptors; visinin staining often filled the cone cell bodies whereas CERN 906 staining tended to localize to the cell membranes (Fig. 1C).

In our next series of experiments, we wanted to determine if the photoreceptors in our cultures were rhythmically secreting melatonin. Photoreceptors are the primary source of melatonin In the retinas of vertebrates [1], [33], [43], [44] and its synthesis in these cells is controlled by the clocks harbored by these cells [1], [4], [45], [46]. We prepared re-aggregate cultures from E6 chicken retinas and exposed them to a 12L:12D cycle for six days. The cultures were then transferred to constant darkness beginning at ZT12 on culture day 7. Robust melatonin secretion rhythms were detected in our cultures beginning on culture day 7 (Fig. 1D, black symbols) with increasing and peak levels of melatonin secretion occuring during the 12 hour dark period. Importantly, this rhythm was retained when the cultures were placed in constant darkness, a result that indicates that the clocks in the photoreceptor cells were actively regulating melatonin secretion from these cells. These findings effectively reproduced those reported by Willbold et al. [37] and extended the analyses of the free-running phase of the constant dark period by an additional 24 hours.

Next, we used these retinal cultures to determine if the melatonin rhythms generated by the photoreceptors could be phase-shifted by light pulses. To test this, we exposed cultures to a 6 hr light pulse beginning at ZT12 on day 7 and then maintained them in constant darkness. Comparisons of the levels of melatonin secreted by these cultures with those secreted by cultures that did not receive the light pulse revealed that there was a dramatic and rapid 6 hr delay in the peak of melatonin release in the cultures exposed to the 6 hr light pulse (Fig. 1D, blue symbols). These results indicate that the biochemical machinery required to phase shift the oscillators that drive photoreceptor melatonin synthesis is in place and is functional.

### Activation of PLC Mimics Light’s Ability to Phase Shift Photoreceptor Melatonin Rhythms

In this series of experiments we set out to determine if activation of PLC in photoreceptors in the absence of light can induce a phase shift in the rhythmic secretion of melatonin that mirrors that induced by a pulse of light. Retinal re-aggregate cultures were exposed to a 12L:12D cycle for six days. At ZT12 on culture day 7, the cultures were treated with 10 µM m-3M3FBS for 6 hr while in the dark and were then kept in constant darkness for the remainder of the experiment.

**Figure 2:**
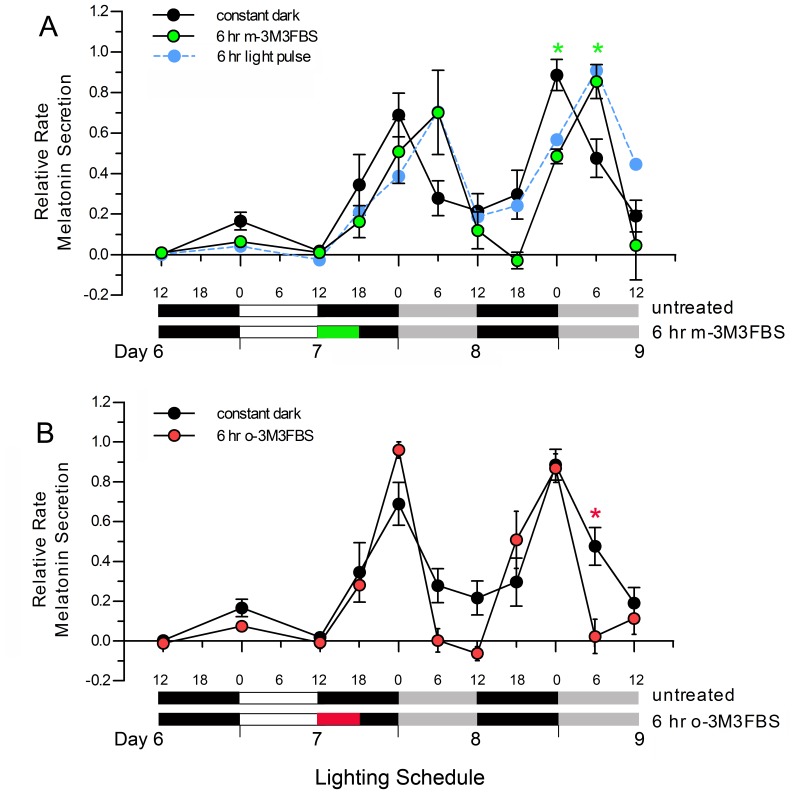
Response of melatonin secretion rhythms in retinal spheroid cultures to activation of PLC. A. Spheroid cultures were exposed to a 12L:12D cycle for six days and were then either placed in constant darkness at ZT12 on culture day 7 (black symbols) or were exposed to 10 µM m-3M3FBS (green rectangle in lighting bar; green symbols) and then placed in constant darkness. The 6 hr light pulse data replotted from Fig. 1D (blue symbols) are shown for reference. B. Spheroid cultures were entrained as described above and were then exposed to a 6 hr pulse of 10 µM o-3M3FBS at ZT12 on culture day 7 (red rectangle in lighting bar; red symbols). Levels of melatonin in the media were measured by competitive ELISA and were used to calculate the relative rates of melatonin secretion from the cultures. Graphs show mean +/− SEM values (untreated, n = 6; light pulse, n = 6; m-3M3FBS, n = 3; o-3M3FBS, n = 3). Time points at which the one-way ANOVA post-hoc tests indicated that there were significant differences (*ρ*<0.05) between the treated and untreated cultures that were maintained in constant darkness are indicated by green asterisks for m-3M3FBS-treated cultures and a red asterisk for o-3M3FBS-treated cultures.

Comparisons of the melatonin secretion rhythms in cultures exposed to the 6 hr pulse of m-3M3FBS (Fig. 2A, green symbols) to those in untreated cultures (Fig. 2A, black symbols) revealed that m-3M3FBS induced a rapid, 6 hr phase delay in the melatonin secretion rhythm that was maintained in constant dark conditions. Importantly, the phase delay induced by the 6 hr exposure to m-3M3FBS was essentially identical to that induced by a comparably timed 6 hr light pulse (Fig. 2A, blue symbols replotted from Fig. 1D). In contrast, the melatonin secretion rhythm in cultures exposed to a 6 hr pulse of o-3M3FBS, the inactive ortho-substituted version of the activator compound (Fig. 2B, red symbols), closely resembled those measured in untreated cultures (Fig. 2B, black symbols).

Analyses of the data collected from the four experimental groups using two-way repeated measures ANOVA revealed that there was a significant group-by-time interaction F(3,14) = P<0.01. Subsequent one-way ANOVAs and post-hoc pairwise comparisons at specific time points revealed that the melatonin secretion rhythms in cultures exposed to 10 µM m-3M3FBS were not significantly different from those exposed to a 6 hr pulse of light; however, both of these groups were significantly different from the untreated cultures at subjective circadian time 0 (CT0) and CT6 on culture day 9 (Fig. 2A, ρ<0.05). Overall, the melatonin secretion rhythms in cultures exposed to o-3M3FBS were very similar to those obtained from untreated cultures (Fig. 2B). We did note that the decline in the rate of melatonin secretion that occurred during the first six hours of subjective light on culture days 8 and 9 was steeper than in the untreated cultures which resulted in a significant difference in secreted melatonin levels at CT6 on culture day 9. The source of this difference is unknown.

## Discussion

Our principal findings are two-fold. First, we demonstrate that the photoreceptors in re-aggregated, spheroid cultures prepared from embryonic chicken retinas exhibit circadian melatonin secretion rhythms that can be dynamically phase shifted by light. Our finding builds on previous observations made by Willbold et al. [37] who showed that the genes encoding arylalkylamine-N-acetyltransferase and hydroxyindole-O-methyltransferase, two key enzymes involved in melatonin synthesis, begin to be expressed in 6-day-old chicken retina spheroids cultures and that the rhythmic secretion of melatonin by these cultures is retained in constant darkness. Our findings extend these observations by showing that a 6 hr pulse of light delivered during the first 6 hours of a 12 hr dark period induces a phase delay in the melatonin secretion rhythm, a result that demonstrates that the light signaling pathways required to phase shift the oscillators that drive melatonin rhythms in the re-aggregate cultures are in place.

Two lines of experimental evidence suggest that the photoreceptor cells are the source of the melatonin rhythms observed in our re-aggregated cultures. Retinal photoreceptors have been shown to be the primary source of rhythmically produced melatonin in chicken [46], rat [4], and *Xenopus* [47] retina; however, at least in the retinas of chickens [31] and rats [48], ganglion cells have also been shown to be able to synthesize melatonin in a rhythmic manner. Of direct relevance to our experiments are the observations in studies of cultured chicken ganglion cells that the amounts and the rhythmic patterns of melatonin release from ganglion cells are quite different from those of photoreceptor cells. Photoreceptors produce approximately 10-fold more melatonin than ganglion cells and the daily rhythms of production of melatonin by these cells are approximately 12 hr out of phase with each other, with the melatonin produced by photoreceptors reaching maximum levels during the 12 hr dark period while that produced by ganglion cells reaches maximum levels during the 12 hr light period [31]. Thus, the pattern and amount of melatonin produced by our cultures is consistent with the photoreceptors being the source of this melatonin.

Our second principal finding is that activation of PLC in the absence of light induces a phase delay in the circadian secretion of melatonin from spheroid cultures that resembles that induced by light. Specifically, we show that exposing retinal spheroid cultures to a 6 hr pulse of m-3M3FBS during the first half of a 12 hr dark period produced a 6 hr phase delay in the melatonin secretion rhythm that is indistinguishable from the phase delay induced by a 6 hr pulse of light delivered to cultures grown in parallel. To our knowledge, this result is the first report showing that PLC activation in and of itself phase shifts photoreceptor rhythms, an observation that fills an important gap in efforts to elucidate the signaling cascade through which light entrains the circadian oscillators in photoreceptors that drive melatonin secretion rhythms.

The results of our experiments suggest that PLC activation is one of the enzymatic steps in the photoreceptor oscillator light entrainment cascade. There are two potential caveats to this conclusion. The first is that m-3M3FBS may be acting through an off target, non-PLC mechanism. Even though m-3M3FBS has been shown to directly activate PLC, it has also has been documented to increase intracellular calcium levels without activating PLC [49], [50]. Our use of the ortho analog, o-3M3FBS, which does not activate PLC at the 10 µM concentration used in our studies [32], provides a measure of control for the off-target effects of m-3M3FBS because o-3M3FBS exhibits many of the same off-target effects exhibited by m-3M3FBS [51]. In our experiments, o-3M3FBS had no effect on the melatonin secretion rhythm generated by our cultures, a result that suggests that m-3M3FBS’s effect on melatonin secretion resulted from activation of PLC. The second caveat stems from the observation that m-3M3FBS is capable of activating a broad spectrum of PLC isoforms [32]. Thus, it is possible that the phase changes that we observed in the melatonin secretion rhythms in response to m-3M3FBS were not caused by activation of a second messenger system specifically linked to activation of PLC. Rather, these phase changes could have occurred in response to activation of a PLC second messenger system that under normal light entraining conditions would be activated by a PLC-independent mechanism.

Our current data do not allow us to rule out these two possibilities; however, experimental evidence from studies of other circadian oscillator cells that support a role for G_q/11_-PLC signaling in mediating the effects of entraining stimuli on the oscillators argues in favor of a similar cascade serving this function in retinal photoreceptors. For example, there is now substantial evidence that intracellular signaling cascade that mediates melanopsin signaling in ipRGCs involves activation of a G_q/11_-type G-protein and subsequent activation of PLC [29]. Of particular interest are the results of studies of light regulation of circadian production of melatonin in cultured chicken retina ganglion cells that suggest that light’s effects on melanopsin production in these cells is likely to be mediated by melanopsin activation of a G_q/11_-PLC signaling cascade in these cells [30], [31], [52], [53]. Further, a recent study of intercellular synchronization of circadian oscillations within the suprachiasmatic nucleus (SCN) clearly demonstrates that activation of G_q_, but not G_s_ or G_i_, in a subset of SCN neurons expressing vasoactive intestinal peptide (VIP) is sufficient and necessary to drive phase changes in the circadian rhythms present in the SCN [54]. While not investigated in this study, it is possible that one of the signaling inputs driving G_q_ activation in SCN VIP neurons and ultimately light entrainment of SCN is the retinohypothalamic tract [55]. Finally, a direct example of the relationship between G_q_ activation and photic entrainment of circadian oscillators was reported in a study of light entrainment of melatonin rhythms in avian pineal gland. This study showed that activation of G_q11_, but not G_i_ or G_o_, produced phase shifts in the melatonin rhythm in pineal that resembled those produced by light [17]. In addition to conservation of G_q_ signaling within these oscillator containing tissues, there is also phyletic conservation of G_q_ signaling as evidenced by the observation that activation of G_q_ drives the entrainment of the various circadian clocks in *Drosophila* [56]. Since G_q_ canonically signals through PLC, it is likely that these circadian inputs are all signaled through PLC, although this has only been rigorously demonstrated for *Drosophila*.

Our findings in the retinal re-aggregate system show that activation of PLC in photoreceptors mimics light’s ability to phase shift melatonin secretion rhythms in photoreceptors. In future studies we will manipulate the PLC signaling cascade in photoreceptors to define PLC’s specific role in mediating the effects of light on the circadian biology of these cells. Defining this signaling pathway in photoreceptors will expand our current understanding of the physiology of these cells and of the neural retina, and may lead to the identification of new candidate genes for photoreceptor diseases. Further, these findings will improve our understanding of the circadian biology of rod and cone cells and identify new benchmarks for evaluating the long-term effectiveness of current therapies designed to treat rod- and cone-specific diseases.
